# FLAIR-Wise Machine-Learning Classification and Lateralization of MRI-Negative ^18^F-FDG PET-Positive Temporal Lobe Epilepsy

**DOI:** 10.3389/fneur.2020.580713

**Published:** 2020-11-03

**Authors:** Iman Beheshti, Daichi Sone, Norihide Maikusa, Yukio Kimura, Yoko Shigemoto, Noriko Sato, Hiroshi Matsuda

**Affiliations:** ^1^Department of Human Anatomy and Cell Science, Rady Faculty of Health Sciences, Max Rady College of Medicine, University of Manitoba, Winnipeg, MB, Canada; ^2^Cyclotron and Drug Discovery Research Center, Southern Tohoku Research Institute for Neuroscience, Koriyama, Japan; ^3^Integrative Brain Imaging Center, National Center of Neurology and Psychiatry, Kodaira, Japan; ^4^Department of Clinical and Experimental Epilepsy, University College London Institute of Neurology, London, United Kingdom; ^5^Department of Radiology, National Center of Neurology and Psychiatry, Kodaira, Japan

**Keywords:** fluid-attenuated inversion recovery, temporal lobe epilepsy, machine-learning, feature extraction, MRI-negative focal epilepsy

## Abstract

**Objective:** In this study, we investigated the ability of fluid-attenuated inversion recovery (FLAIR) data coupled with machine-leaning algorithms to differentiate normal and epileptic brains and identify the laterality of focus side in temporal lobe epilepsy (TLE) patients with visually negative MRI.

**Materials and Methods:** The MRI data were acquired on a 3-T MR system (Philips Medical Systems). After pre-proceeding stage, the FLAIR signal intensities were extracted from specific regions of interest, such as the amygdala, cerebral white matter, inferior temporal gyrus, middle temporal gyrus, parahippocampal gyrus, superior temporal gyrus, and temporal pole, and fed into a classification framework followed by a support vector machine as classifier. The proposed lateralization framework was assessed in a group of MRI-negative unilateral TLE patients (*N* = 42; 23 left TLE and 19 right TLE) and 34 healthy controls (HCs) based on a leave-one-out cross-validation strategy.

**Results:** Using the FLAIR data, we obtained a 75% accuracy for discriminating the three groups, as well as 87.71, 83.01, and 76.19% accuracies for HC/right TLE, HC/left TLE, and left TLE/right TLE tasks, respectively.

**Interpretation:** The experimental results show that FLAIR data can potentially be considered an informative biomarker for improving the pre-surgical diagnostic confidence in patients with MRI-negative TLE.

## Highlights

- We assessed the performance of FLAIR data for classifying MRI-negative TLE.
- Our prediction model showed 75% accuracy for discriminating the three groups.
- FLAIR data are a potential biomarker for MRI-negative TLE classification tasks.

## Introduction

Temporal lobe epilepsy (TLE), the most common focal epilepsy in adults, has good surgical outcomes despite its association with drug resistance (1). The main role for structural magnetic resonance imaging (MRI) in clinical epileptology lies in detection of etiology, location of the epileptogenic zone and focal epileptogenic lesions (2). However, about 30% of people with TLE show no abnormalities or epileptogenic lesions on conventional MRI (3). This type of TLE patient has visually normal brain MRI and is said to have “MRI-negative TLE.” Functional imaging techniques such as positron emission tomography (PET) and single-photon emission computed tomography (SPECT) are powerful modalities for identifying and monitoring patients with TLE when MRI is visually negative (3, 4). Indeed, interictal ^18^F-FDG PET achieves 85–90% sensitivity in TLE lateralization (5). Additionally, when ^18^F-FDG PET provides positive findings in MRI-negative TLE, surgical treatment is associated with favorable prognosis in patients with hippocampal sclerosis (6–8). Thus, MRI-negative PET-positive TLE is also considered an important group with favorable surgical outcomes. However, as a common drawback, the practical utility of functional imaging techniques such as PET and SPECT is restricted by infrastructure costs and poor access to PET or SPECT scanners in many hospitals, particularly in developing countries (9). Additionally, nuclear imaging always necessitates radiation exposure. Therefore, advanced MRI analysis is expected to become a widely available and less invasive tool. Indeed, some neuroimaging studies have shown that the FLAIR modality might be useful for detecting lesions in patients with focal epilepsy (10). Because FLAIR signal abnormality is sometimes found in epileptogenic lesions and astrogliosis (2, 11), the optimal pattern classification of FLAIR signals may provide significant clinical lateralization in patients with visually MRI-negative TLE.

To overcome shortcomings, we analyzed the utility of FLAIR imaging as an alternative modality for solving the classification and lateralization problem in MRI-negative TLE patients. We hypothesized that FLAIR data coupled with machine-learning algorithms would allow us to distinguish TLE patients from healthy controls (HCs) and determining the lateralization of MRI-negative TLE individuals. To this end, we applied a machine-learning algorithm to the FLAIR signal intensity data extracted from specific regions of interest (ROIs) such as the amygdala, cerebral white matter, inferior temporal gyrus, middle temporal gyrus, parahippocampal gyrus, superior temporal gyrus, and temporal pole. We assessed the efficiency of the proposed methodology in 19 MRI-negative right TLE (RTLE) patients, 23 MRI-negative left TLE (LTLE) patients, and 34 HCs by means of a leave-one-out cross-validation (LOOCV) strategy and with a support vector machine (SVM) as classifier.

## Materials and Methods

### Study Population

**Figure 1:**
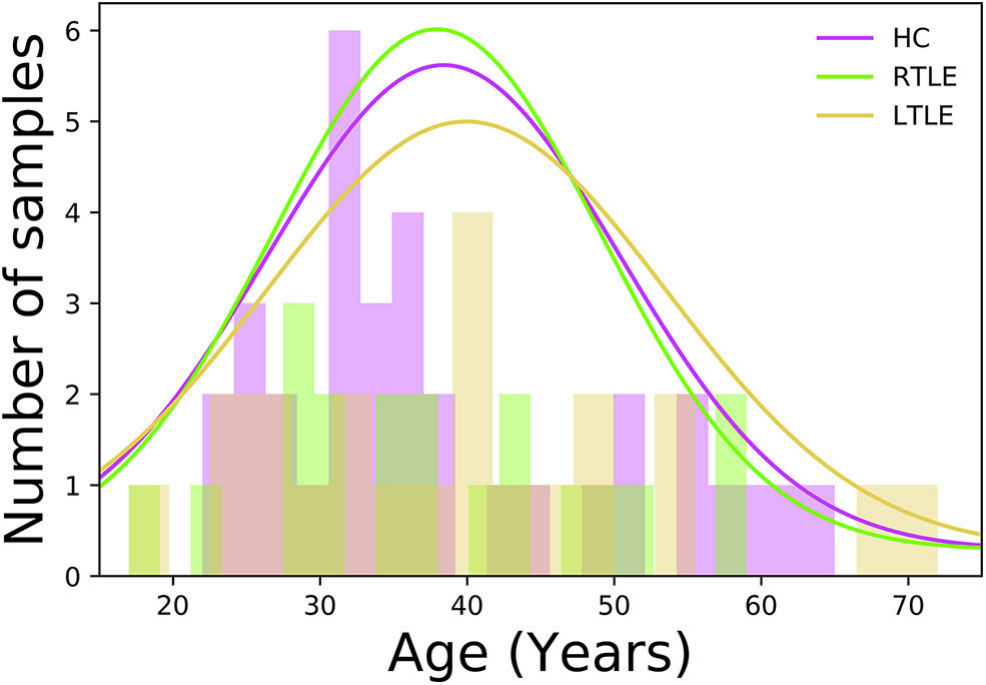
Histogram displaying the age distribution among different groups.

In this study, a total of 76 individuals−34 HCs (50% female, 38.4 ± 12.1 years old, range 22–65 years) and 42 MRI-negative PET-positive unilateral TLE patients [23 LTLE (34.7% female, 39.0 ± 14.3 years old, range 17–72 years) and 19 RTLE (57.8% female, 37.9 ± 11.4 years old, range 17–59 years)]—were recruited at the National Center of Neurology and Psychiatry (NCNP) Hospital, Tokyo, Japan, between January 2015 and November 2017. Regarding age at examination, there were no significant differences among three groups (*F* = 0.04, *p* = 0.96; ANOVA). Figure 1 displays the distribution of age at examination among different groups. All participants provided written informed consent, and this study was approved by the Institutional Review Board at the National Center of Neurology and Psychiatry Hospital, Tokyo, Japan.

### Visual Assessment of MRI and FDG PET

Experienced neuroradiologists visually assessed the MRI and FDG PET scans, with clinical information of patients. Regarding MRI, hippocampal atrophy, signal changes, and loss of internal structures were carefully excluded. To rule out visually-detectable focal cortical dysplasia, we carefully checked increased cortical thickness, blurring of the gray-white matter interface, and abnormal hyperintensity in FLAIR and T2-weighted images. We also excluded other possible epileptogenic lesions, e.g., tumors, vascular malformation, and encephaloceles, carefully. For visual analyses of FDG PET, the reviewer decided whether unilateral hypometabolism was found or not by comparing left and right temporal and extratemporal lobes.

### Image Acquisition and Pre-processing

MRI for all patients was performed on a 3-T MR system with a 32-channel coil (Philips Medical Systems, Best, The Netherlands). The sequences and their parameters were as follows: three-dimensional (3D) sagittal T1-weighted magnetization prepared rapid acquisition with gradient echo (MPRAGE) images [repetition time (TR)/echo time (TE), 7.18 ms/3.46 ms; flip angle, 10°; 0.6-mm effective slice thickness with no gap; 300 slices; matrix, 384 × 384; field of view (FOV), 26.1 × 26.1 cm]; sagittal 3D fluid-attenuated inversion recovery (FLAIR) images (TR/TE, 4700/283 ms; inversion time, 1,600 ms; thickness, 0.55 mm with no gap; 340 slices; matrix, 512 × 465; FOV, 26.0 × 23.4 cm). Additionally, for visual assessment of potential epileptogenic lesions, both T1 and FLAIR images were reconstructed into coronal and axial slices and high-resolution coronal T2-weighted images were also obtained. The FLAIR scans were analyzed by Statistical Parametric Mapping toolbox version 12 (http://www.fil.ion.ucl.ac.uk/spm/software/spm12/). First, for each individual, we registered the FLAIR scan to the respective high-resolution MRI scan on the basis of linear affine transformation (SPM12 toolbox; coregister fuction, default setting, 4th degree B-spline tri-linear interpolation method and normalized mutual information cost function). Then, we applied a spatially adaptive non-local means denoising filter to the FLAIR scans through the CAT12 toolbox (http://www.neuro.uni-jena.de/cat/). Afterward, we applied a special normalization to the bias-corrected FLAIR scans using the Clinical toolbox (https://www.nitrc.org/projects/clinicaltbx/), followed by a default FLAIR template generated from 181 individuals (56% female, 39.9 ± 9.3 years old, range 26–76 years, available at https://brainder.org/download/flair/) (12). As for the feature extraction stage, we extracted the average FLAIR signal intensities from specific ROIs. We selected the ROIs of temporal lobe gray matters and cerebral white matters, which may reflect potential epileptogenic lesions, astrogliosis, and related changes. Specifically, the following ROIs were selected using the SPM template: the amygdala, cerebral white matter, inferior temporal gyrus, middle temporal gyrus, parahippocampal gyrus, superior temporal gyrus, and temporal pole. The mean FLAIR signal intensities extracted from specific ROIs were considered a feature for classification models.

### Validation and Classification Performance

To validate the performance of FLAIR data for the diagnosis and lateralization of MRI-negative ^18^F-FDG PET-positive TLE, we built three binary classification models (i.e., HC/RTLE, HC/LTLE, and RTLE/LTLE) as well as a multiclass classification model (i.e., HC/RTLE/LTLE). The performance of each classification model was computed using a LOOCV strategy such that, each time, one sample was used for the test set and the remainder for training. A SVM implemented in MATLAB (i.e., “fitcsvm” function, linear kernel, default set of parameters) was used a classifier. Using a linear SVM kernel, we are able to visualize the contribution of individual feature (i.e., region) to the overall classification models. As for binary classification models, the prediction performance was assessed by means of accuracy (ACC), sensitivity (SEN), specificity (SPE), and area under the receiver operating characteristic curve (ROC-AUC) metrics, whereas the classification performance for the multiclass classification model was reported in terms of accuracy and confusion matrix. In addition, to determine the contribution of each feature (i.e., region) in our classification models, we aggregated the SVM coefficients achieved from training sets over the LOOCV strategy and then averaged them.

## Experimental Results

### Clinical Assessments

**Figure 2:**
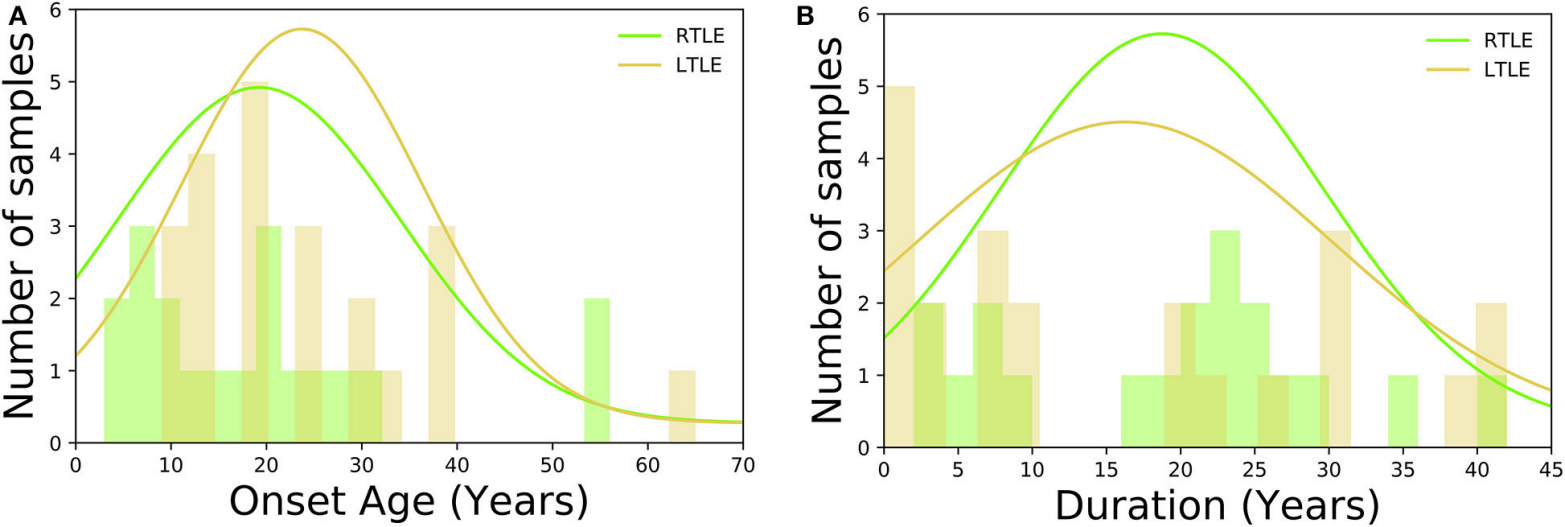
Histogram displaying the clinical demographics among TLE subjects: **(A)** onset age, **(B)** duration of disease.

As for HC group, participants had no history of neurological or psychiatric diseases, no use of medication affecting the central nervous system, and no structural abnormalities on MRI. TLE was diagnosed based on the presence of focal seizures consistent with TLE and focal epileptiform discharge predominantly in temporal areas on a conventional scalp electroencephalogram (EEG) using standard international 10–20 system. All patients underwent 3-T MRI and interictal FDG PET, which were visually evaluated by experienced neuroradiologists. All TLE patients showed unilateral glucose hypometabolism including the temporal lobe in interictal FDG PET consistent with the clinical symptoms and EEG abnormalities, without any evidence of morphological abnormality on visual assessment of MRI scans. To investigate a distinct group of MRI-negative PET-positive unilateral TLE, we recruited only patients with concordant ipsilateral hypometabolism in FDG PET, and all TLE patients were drug-resistant. In TLE patients' group, there were no significant differences between RTLE and LTLE in terms of onset age (RTLE = 19.2 ± 15.2 years; LTLE = 23.3 ± 12.7 years; *t*-test = 0.94, *p* = 0.34; student *t*-test) and duration of disease (RTLE = 18.7 ± 11.1 years; LTLE = 15.6 ± 14.2 years; *t*-test = −0.75, *p* = 0.45; student *t*-test). Figure 2 displays the distribution of onset age, and duration of disease related to TLE subjects used in this study. The detailed clinical demographics related to TLE patients is presented in Table 1. A few patients showed bilateral interictal epileptiform discharges, but long-term video-EEG confirmed the laterality of them. Of total 42 patients, five cases (No. 9, 22, 25, 31, and 38) underwent resection surgery and focal cortical dysplasia was confirmed in all patients except one case with gliosis (No. 22). Additionally, the details of extended hypometabolic areas in visual assessment of FDG PET in each patient are shown in Table 1.

**Table 1:** Clinical demographics and results of exams in patients with TLE.

**No**.	**Age (range)**	**sex**	**onset age**	**Duration of TLE**	**Focus side**	**Seizure type**	**IED location**	**Anti-epileptic drugs**	**LT-VEEG**	**Hypometablic areas in FDG-PET**
1	55–59	F	13	42	L-TLE	FAS, FIAS	T3	CBZ, TPM, PRM	Yes	LT, OL, IFG
2	45–49	M	9	39	L-TLE	FAS, FIAS	T3	LEV, PRM, CBZ	No	MT, LT, OL
3	45–49	M	29	19	L-TLE	FIAS	T3	CBZ, LTG	No	MT, LT
4	40–44	M	9	31	L-TLE	FIAS	T3	CBZ, VPA, LCM, CZP	No	MT, LT
5	40–44	M	20	23	L-TLE	FIAS	T3	CBZ, LEV	No	MT, LT
6	25–29	F	19	8	L-TLE	FIAS	T3	CBZ, TPM	Yes	LT, OL, OFC
7	70–74	M	65	7	L-TLE	FAS, FIAS	T3	CBZ	No	MT
8	25–29	M	19	9	L-TLE	FAS, FIAS	T3, T4	CBZ, LEV, CLB	Yes	LT, MT
9	30–34	M	24	8	L-TLE	FIAS	T3	CBZ, LTG, CZP	Yes	MT
10	45–49	F	20	27	L-TLE	FIAS	T3	CBZ, CZP	Yes	MT
11	40–44	M	32	9	L-TLE	FIAS	F7	LEV, CZP, ZNS, CLB	No	LT, MT, IFG
12	15–19	F	14	3	L-TLE	FIAS	T3	CBZ, LEV	Yes	MT
13	35–39	M	37	1	L-TLE	FAS, FIAS	T3, T5	LEV	No	MT
14	25–29	F	25	2	L-TLE	FIAS	T3	CBZ	Yes	MT, LT
15	40–44	M	11	30	L-TLE	FAS, FIAS, FBTCS	F7	PHT, CBZ	Yes	LT, MT, OL
16	30–34	F	14	20	L-TLE	FIAS, FBTCS	T3	CBZ, ZNS	Yes	LT, OL
17	20–24	M	24	0	L-TLE	FIAS	T3	LEV, VPA	No	LT, MT, OL
18	65–69	M	37	30	L-TLE	FIAS	T3	VPA, CBZ	No	MT, LT
19	40–44	F	38	2	L-TLE	FIAS, FBTCS	T3, T4	CBZ	Yes	MT, LT
20	50–54	M	13	41	L-TLE	FAS, FIAS	T3	CBZ	No	MT, LT
21	30–34	F	30	2	L-TLE	FAS, FIAS	T3	LEV	Yes	LT
22	20–24	M	20	4	L-TLE	FIAS, FBTCS	F7, T3	VPZ, CBZ	Yes	LT, MT, OFC, OL
23	15–19	M	14	3	L-TLE	FAS, FIAS	T1	ZNS, LEV	Yes	MT, LT
24	45–49	M	26	21	R-TLE	FIAS	T4	CBZ, VPA	No	MT, LT
25	50–54	F	22	29	R-TLE	FIAS	T4, T3	CBZ	Yes	LT
26	30–34	M	15	16	R-TLE	FAS, FIAS	T4	LEV, CBZ, PHT	No	MT
27	45–49	F	7	42	R-TLE	FIAS	T4	CBZ, VPA, CLB	No	LT, IFG, operculum
28	25–29	M	6	22	R-TLE	FAS, FIAS	T4	CBZ, LTG, CZP	Yes	MT, LT, IFG, operculum
29	30–34	F	6	25	R-TLE	FAS, FIAS	F8	CBZ	Yes	LT
30	55–59	M	54	5	R-TLE	FIAS	F8	ZNS, LEV	No	MT
31	15–19	M	11	6	R-TLE	FAS, FIAS	T4, T6	PHT, LEV, CBZ	Yes	MT, LT, OL
32	25–29	M	9	19	R-TLE	FAS, FIAS	T3, T4	PHT, LEV, TPM	Yes	MT, LT
33	40–44	F	20	23	R-TLE	FAS, FIAS	T4	CBZ	No	LT, MT, OL
34	25–29	F	20	8	R-TLE	FAS, FIAS, FBTCS	F8, T4	VPA, CBZ	Yes	MT, LT
35	35–39	M	10	27	R-TLE	FAS, FIAS	F8, F7	CBZ, LEV	Yes	LT, OL
36	30–34	M	32	2	R-TLE	FIAS, FBTCS	F8	CBZ, LEV	No	LT
37	35–39	F	28	7	R-TLE	FAS, FIAS, FBTCS	T4	CBZ	No	LT
38	35–39	F	3	34	R-TLE	FAS, FIAS	T4	CBZ, VPA	Yes	LT
39	40–44	F	20	23	R-TLE	FIAS	T4	CBZ, VPA, CLB	Yes	MT, LT, OFC
40	40–44	F	17	25	R-TLE	FIAS	T4	PHT, LTG	No	LT, MT, OL, operculum
41	55–59	F	56	2	R-TLE	FIAS	T3	VPA	No	MT, LT
42	20–24	F	3	20	R-TLE	FAS, FIAS	T4	LEV, CBZ, PHT	Yes	MT, LT

*CBZ, carbamazepine; CLB, clobazam; CZP, clonazepam; EEG, Electroencephalography; FAS, focal aware seizures; FBTCS, focal to bilateral tonic-clonic seizures; FIAS, focal impaired awareness seizures; IED, interictal epileptiform discharge; IFG, inferior frontal gyrus; LCM, lacosamide; LEV, levetiracetam; LT, lateral temporal lobe; LT-VEEG, long-term video-EEG monitoring; LTG, lamotrigine; PHT, phenytoin; PRM, primidone; MT, mesial temporal lobe; NZP, nitrazepam; OFC, orbitofrontal cortex; OL, occipital lobe; TPM, topiramate; VPA, valproate; ZNS, zonisamide*.

### Experimental Results

**Figure 3:**
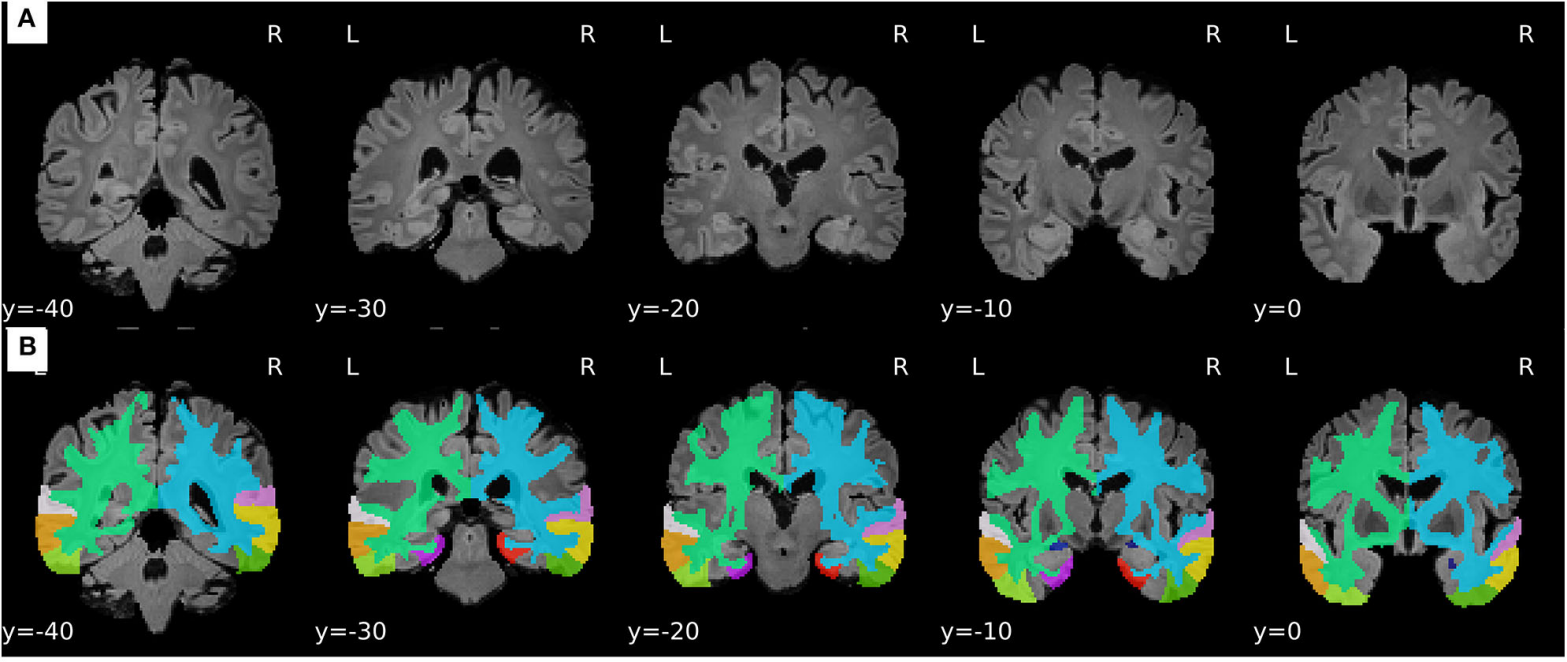
Representative samples of the pre-processing stage and feature extraction from FLAIR images. **(A)** Processed FLAIR images with a resolution of 1.5 × 1.5 × 1.5 mm. **(B)** Binary masks defined based on the selected ROIs.

**Figure 4:**
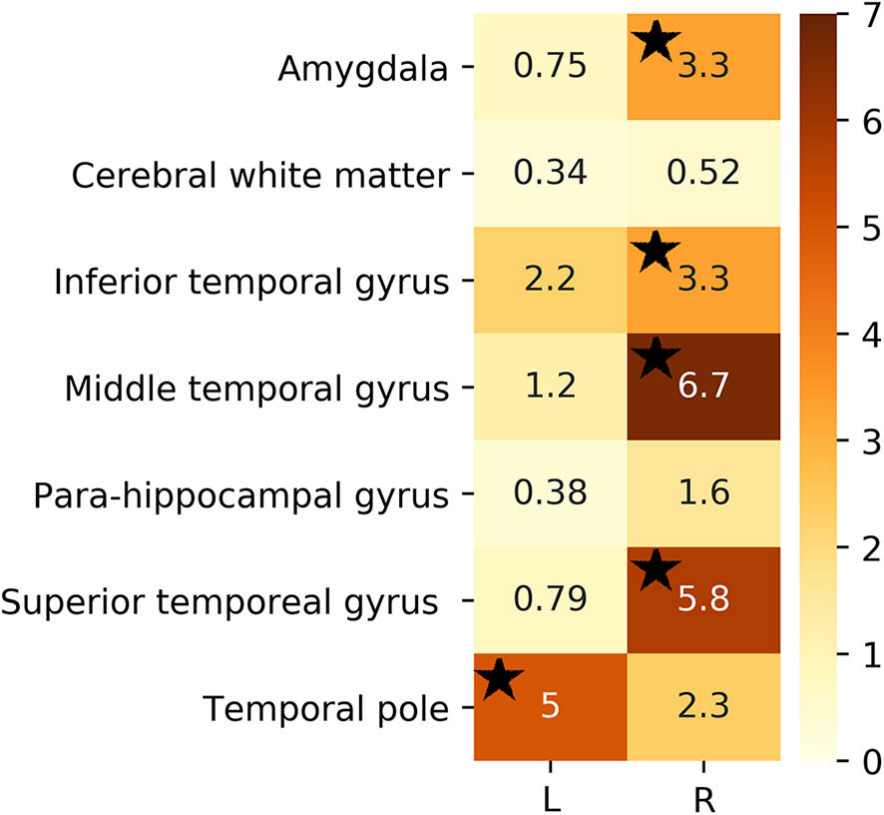
*F*-test value of extracted FLAIR data from selected ROIs among the three groups. Black stars denote regions that were significant (*p* < 0.05).

For the sake of illustration, Figure 3 shows representative FLAIR images and the binary masks for extracting the FLAIR signal intensities from specific regions. A summary of statistical analysis (i.e., ANOVA test) of FLAIR data extracted from the specific regions among the three groups is displayed in Figure 4. There was significant difference in term of FLAIR data for right amygdala, right inferior temporal gyrus, right middle temporal gyrus, right superior temporal gyrus, and left temporal pole regions among the three groups (*p* < 0.05, ANOVA).

**Figure 5:**
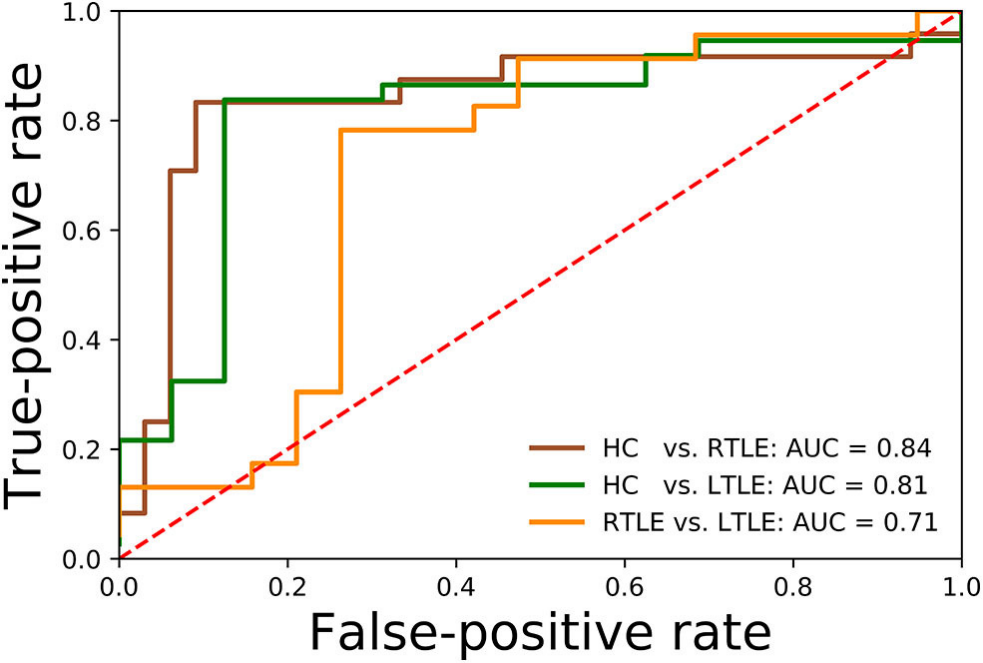
ROCs related to the binary classification models.

**Figure 6:**
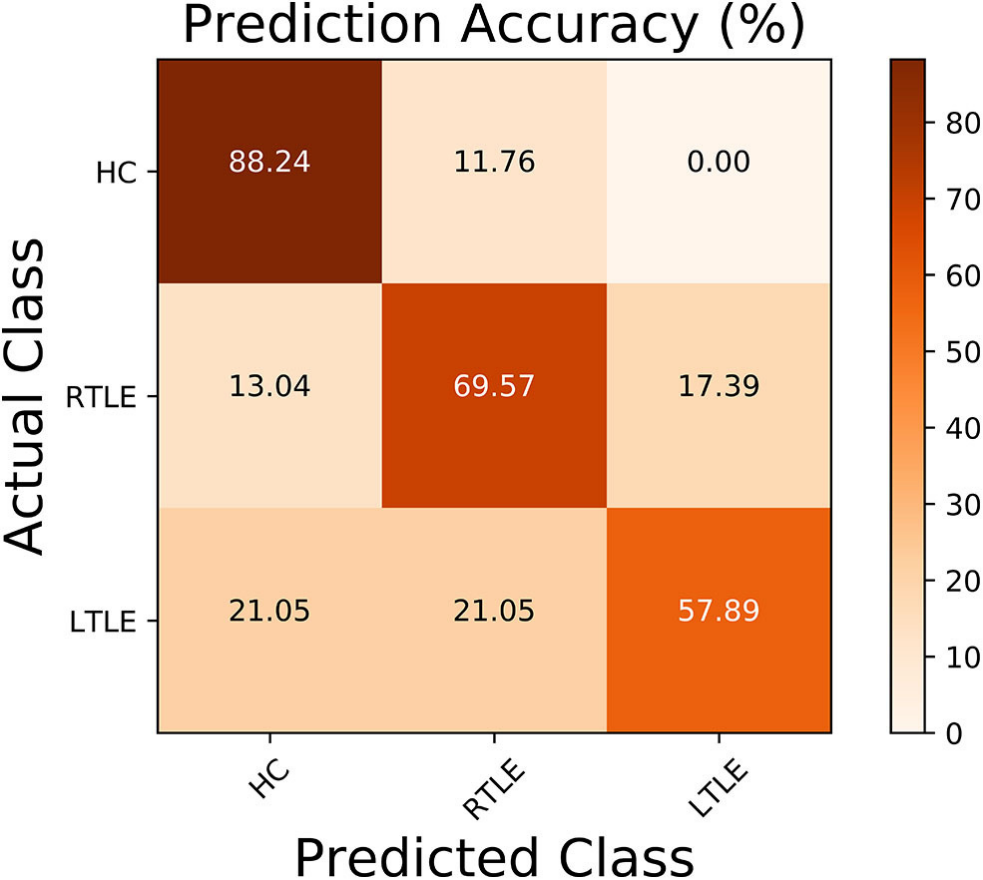
Normalized confusion matrix related to the multiclass classification model.

**Figure 7:**
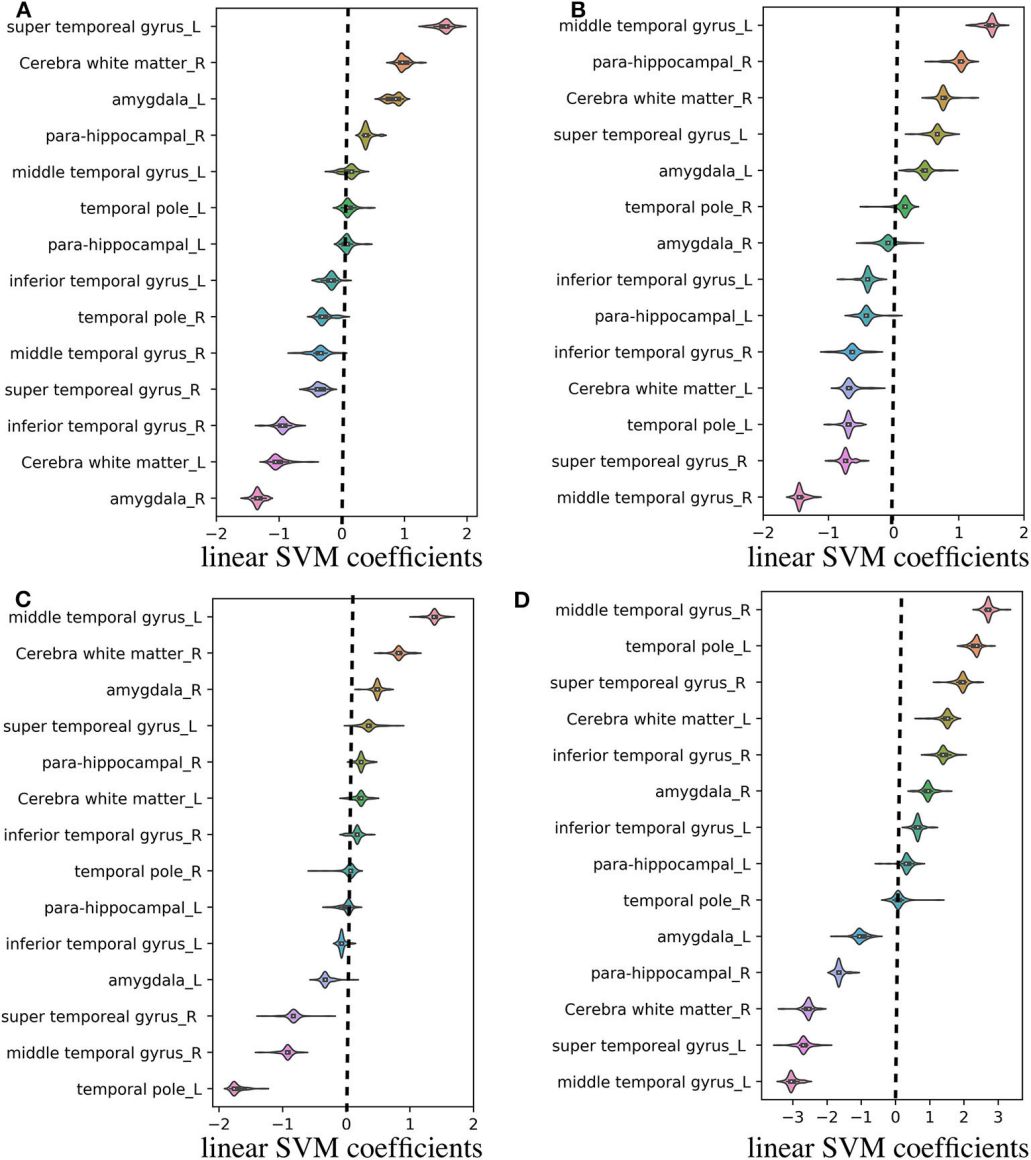
Violin plot of feature (i.e., region) contributions for each classification model. **(A)** RTLE/LTLE, **(B)** HC/LTLE, **(C)** HC/RLTE, and **(D)** HC/RTLE/LTLE. For each prediction model, the linear SVM coefficients were computed through all LOOCV iterations. Regarding the binary classification models, features (i.e., regions) with positive and negative coefficients stand for positive and negative correlations with probability of classification, respectively. As for the multiclass classification model, the linear SVM coefficients was achieved by linear combination of three binary learners through “fitcsvm” function.

Regarding the binary classification models, HCs were distinguished from RTLE patients with 87.71% accuracy (SEN 86.95%, SPE 88.23%, and AUC 0.84) and from LTLE patients with 83.01% accuracy (SEN 91.17%, SPE 68.42%, and AUC 0.81) while LTLE patients were differentiated from RTLE patients with 76.19% accuracy (SEN 78.26%, SPE 73.26%, and AUC 0.71). Figure 5 shows the ROC curves for the binary classification models. Regarding the multiclass classification task for our dataset, we obtained a prediction accuracy of 75% for discriminating among the three groups (i.e., HC/RTLE/LTLE). Figure 6 illustrates the confusion matrix of predicted labels against actual labels for the multiclass classification model. Figure 7 shows the ranking of features (i.e., regions), which contributed to classification models on the basis of the linear SVM coefficients.

### Effect of Hippocampus on Prediction Models

To assess the impact of hippocampus on the classification performances, we recomputed the prediction accuracies after adding the hippocampus data in our prediction models. The classification results were as follow: HC/RTLE = 87.71%, HC/LTLE = 79.24%, RTLE/LTLE = 71.42%, and HC/RTLE/LTLE = 69.73%. By comparing these results with prediction results presented in section “Experimental Results”, it can be seen that including the hippocampus data in prediction models could not improve the prediction accuracies for distinguishing TLE patients from HCs and detecting the lateralization of MRI-negative TLE individuals.

### Results of Whole Brain FLAIR Data and Data Reduction

In this study, we selected the specific ROIs in accordance with the pathology-based knowledge which may reflect potential epileptogenic lesions, astrogliosis, and related changes (i.e., pre-defined regions-based strategy). To show whether the selected regions were appropriate for FLAIR-wise classification and lateralization models, we validated the classification models using the whole-brain FLAIR signal intensities (in total, 136 regions). In this case (i.e., using whole-brain FLAIR data), the performance of our classification models in terms of accuracy were as follows: HC/RTLE = 82.45%, HC/LTLE = 75.47%, RTLE/LTLE = 54.76%, and HC/RTLE/LTLE = 56.57%. Furthermore, we applied principal component analysis (PCA) data reduction technique on whole-brain FLAIR data. As for PCA data reduction followed by 95% amount of total variance, we achieved the following prediction results: HC/RTLE = 75.43%, HC/LTLE = 73.58%, RTLE/LTLE = 42.58%, and HC/RTLE/LTLE = 50%. When we compared the prediction results using whole-brain FLAIR signal intensities, PCA data reduction method, and FLAIR signal intensities extracted from specific regions (see section “Experimental Results”), it was evident that our pre-defined regions-based strategy might provide more informative data for the classification and lateralization of MRI-negative TLE individuals rather than whole-brain FLAIR data and PCA data reduction.

## Discussion

A series of neuroimaging studies have designed and developed new techniques for the classification and lateralization of TLEs based on different brain imaging modalities. For instance, the researchers in (13) suggested a lateralization model based on a multimodality dataset (i.e., T1 images, T2 images, fractional anisotropy, and mean diffusivity) to classify left-sided seizure patients from right-sided patients. They assessed the reliability of their model in 17 TLE patients (9 in the left-sided seizure onset group and 8 in the right-sided seizure onset group) and reported an accuracy of 100% for left-sided seizure and 88.9% for right-sided seizure. Another study was conducted on a lateralization model based on structural MRI and diffusion tensor data (14) followed by a sparse linear regression feature-selection method. They achieved a lateralization accuracy from 72.7 to 86.4% by integrating the structural MRI data with diffusion tensor data. It should be noted that most of these studies were conducted using TLE patients who showed morphological abnormalities on MRI scans. Although PET or SPECT images can be considered powerful modalities for tracing and monitoring epilepsy in MRI-negative patients, these functional imaging techniques require high infrastructure costs for implementation and are not accessible in all hospitals, particularly those in developing countries.

Lateralizing the focus side of TLE is a highly relevant in drug-resistant patients. According to the previous studies (6, 8), MRI-negative PET-positive TLE showed good postsurgical seizure freedom mostly after standard anterior temporal lobectomy. Thus, although this study lacked the detailed epileptogenic zone or surgical outcome, we consider that lateralization plays a key role in clinical practice, given the favorable outcome by the established surgical method. In this regard, interictal FDG PET is an established tool with 85–90% sensitivity for TLE lateralization (5).

Accordingly, we were motivated to assess the utility of FLAIR data, as a low-cost and widely available modality, coupled with machine-learning algorithms for addressing the classification and lateralization problem in MRI-negative TLE patients. To this end, we extracted the FLAIR signals from specific parts of brain (i.e., the amygdala, cerebral white matter, inferior temporal gyrus, middle temporal gyrus, parahippocampal gyrus, superior temporal gyrus, and temporal pole) and then fed the data into the classification models. Using this strategy, our experimental results determined that FLAIR data can distinguish the three groups (i.e., HC/RTLE/LTLE) with 75% accuracy, suggesting that FLAIR data has the potential to be considered a lower-cost alternative for the classification and lateralization of MRI-negative TLE patients.

Based on the information obtained in the machine-learning stage, we specified the contribution of each region for the classification models (see Figure 7). This visualization may be useful for the clinical application of quantitative FLAIR signal analysis to the lateralization of the focal epileptogenic lesions in TLE, although further external validation is needed. Based on linear SVM coefficients achieved from machine learning stage, most of the relevant gray matter regions within the temporal lobe exhibited remarkable contributions in the prediction models, including the temporal pole, which is the most frequent location of epileptogenicity in MRI-negative PET-positive TLE (8). Moreover, it should be noted that the cerebral white matter was also important, given that FLAIR hyperintensity in the ipsilateral white matter has been repeatedly reported, particularly in the anterior temporal lobe, in TLE (11, 15). Although the causes and clinical effects of this T2/FLAIR hyperintensity had been controversial, a strong evidence suggested an association with degeneration of fiber bundles and axonal damages (11, 15, 16).

In the field of epilepsy neuroimaging, some studies have documented an important link between the hippocampus and epilepsy, particularly in TLE patients with hippocampal sclerosis (17). However, a recent study has reported that features in the hippocampus appear to be less important than those in the temporal lobe in MRI-negative TLE populations (18). To elucidate on the underlying mechanism hippocampus on the classification performances, we added hippocampus data along with other selected ROIs in our prediction models. However, when we considered the hippocampus in the list of selection regions, our experimental results showed no improvement in the prediction accuracies in our classification models (section “Effect of Hippocampus on Prediction Models”). We therefore did not retain the hippocampus region in our classification models. One explanation might be that the hippocampal FLAIR signal is not informative in MRI-negative TLE because the focus is more likely to be located in cortical regions. As for the hippocampus region, our machine-learning based results are in agreement with findings from (18).

To our knowledge, this is the first study to use pattern analysis of FLAIR data for the classification and lateralization of MRI-negative TLE patients. Future research into the use of FLAIR markers for the diagnosis and lateralization of MRI-negative PET-positive TLE individuals is nonetheless required. Finally, the lack of detailed location of definite epileptogenic zone and surgical outcome is an important limitation of this study. On the other hand, given the evidence on the good seizure outcome after anterior temporal lobectomy in MRI-negative PET-positive TLE (6, 8), it is expected that FDG PET could potentially be an alternative indicator of focus side, and therefore we focused on lateralization by FDG PET.

## Conclusion

The aim of this study was to examine the utility of FLAIR data for the classification and lateralization of TLE patients with visually normal brain MRIs. Our experimental results indicated that the FLAIR modality has remarkable functionality and could be considered an accessible, safe, low-cost, and informative predictor for the classification and lateralization of MRI-negative TLE individuals in the clinical setting.

## Data Availability Statement

The data analyzed in this study is subject to the following licenses/restrictions: It is a private dataset. Requests to access these datasets should be directed to Iman Beheshti, beheshtiiman@gmail.com.

## Ethics Statement

The studies involving human participants were reviewed and approved by all participants provided written informed consent, and the study was approved by the Institutional Review Board at the National Center of Neurology and Psychiatry Hospital. The patients/participants provided their written informed consent to participate in this study.

## Author Contributions

IB and DS designed research and performed research and wrote the paper. NM, YK, YS, and NS collected the data and performed PET interpretation. HM supervised the study. All authors contributed to the article and approved the submitted version.

## Conflict of Interest

The authors declare that the research was conducted in the absence of any commercial or financial relationships that could be construed as a potential conflict of interest.

## References

[1] Wiebe S Blume WT Girvin JP Eliasziw M A randomized, controlled trial of surgery for temporal-lobe epilepsy. N Engl J Med. (2001) 345:311–8. 10.1056/NEJM200108023450501 11484687

[2] Bernasconi A Cendes F Theodore WH Gill RS Koepp MJ Hogan RE Recommendations for the use of structural magnetic resonance imaging in the care of patients with epilepsy: a consensus report from the International League Against Epilepsy Neuroimaging Task Force. Epilepsia. (2019) 60:1054–68. 10.1111/epi.15612 31135062

[3] Muhlhofer W Tan Y Mueller SG Knowlton R MRI-negative temporal lobe epilepsy—what do we know? Epilepsia. (2017) 58:727–42. 10.1111/epi.13699 28266710

[4] Kotikalapudi R Martin P Marquetand J Lindig T Bender B Focke NK. Systematic assessment of multispectral voxel-based morphometry in previously MRI-negative focal epilepsy. Am J Neuroradiol. (2018) 39:2014–21. 10.3174/ajnr.A5809 30337431 PMC7655351

[5] Kumar A Chugani HT. The role of radionuclide imaging in epilepsy, part 1: sporadic temporal and extratemporal lobe epilepsy. J Nucl Med. (2013) 54:1775–81. 10.2967/jnumed.112.114397 23970368

[6] LoPinto-Khoury C Sperling MR Skidmore C Nei M Evans J Sharan A. Surgical outcome in PET-positive, MRI-negative patients with temporal lobe epilepsy. Epilepsia. (2012) 53:342–8. 10.1111/j.1528-1167.2011.03359.x 22192050

[7] Chassoux F Rodrigo S Semah F Beuvon F Landre E Devaux B. FDG-PET improves surgical outcome in negative MRI Taylor-type focal cortical dysplasias. Neurology. (2010) 75:2168–75. 10.1212/WNL.0b013e31820203a9 21172840

[8] Kuba R Tyrlíková I Chrastina J Slaná B PaŽourková M Hemza J. MRI-negative PET-positive” temporal lobe epilepsy: invasive EEG findings, histopathology, and postoperative outcomes. Epilepsy Behav. (2011) 22:537–41. 10.1016/j.yebeh.2011.08.019 21962756

[9] Kashyap R Dondi M Paez D Mariani G. Hybrid imaging worldwide—challenges and opportunities for the developing world: a report of a technical meeting organized by IAEA. in Semin Nucl Med. (2013) 43:208–23. 10.1053/j.semnuclmed.2013.02.001 23561459

[10] Focke NK Symms MR Burdett JL Duncan JS. Voxel-based analysis of whole brain FLAIR at 3T detects focal cortical dysplasia. Epilepsia. (2008) 49:786–93. 10.1111/j.1528-1167.2007.01474.x 18076641

[11] Adachi Y Yagishita A Arai N. White matter abnormalities in the anterior temporal lobe suggest the side of the seizure foci in temporal lobe epilepsy. Neuroradiology. (2006) 48:460–4. 10.1007/s00234-006-0092-1 16645843

[12] Rorden C Bonilha L Fridriksson J Bender B Karnath H-O. Age-specific CT and MRI templates for spatial normalization. Neuroimage. (2012) 61:957–65. 10.1016/j.neuroimage.2012.03.020 22440645 PMC3376197

[13] Cantor-Rivera D Khan AR Goubran M Mirsattari SM Peters TM. Detection of temporal lobe epilepsy using support vector machines in multi-parametric quantitative MR imaging. Comput Med Imaging Graph. (2015) 41:14–28. 10.1016/j.compmedimag.2014.07.002 25103878

[14] Kamiya K Amemiya S Suzuki Y Kunii N Kawai K Mori H. Machine learning of DTI structural brain connectomes for lateralization of temporal lobe epilepsy. Magn Reson Med Sci. (2016) 15:121–9. 10.2463/mrms.2015-0027 26346404

[15] Casciato S Picardi A D'Aniello A De Risi M Grillea G Quarato PP. Temporal pole abnormalities detected by 3 T MRI in temporal lobe epilepsy due to hippocampal sclerosis: no influence on seizure outcome after surgery. Seizure. (2017) 48:74–8. 10.1016/j.seizure.2017.04.006 28431291

[16] Mitchell LA Jackson GD Kalnins RM Saling MM Fitt GJ Ashpole RD. Anterior temporal abnormality in temporal lobe epilepsy: a quantitative MRI and histopathologic study. Neurology. (1999) 52:327. 10.1212/WNL.52.2.327 9932952

[17] Beheshti I Sone D Farokhian F Maikusa N Matsuda H. Gray matter and white matter abnormalities in temporal lobe epilepsy patients with and without hippocampal sclerosis. Front Neurol. (2018) 9:107. 10.3389/fneur.2018.00107 29593628 PMC5859011

[18] Bennett OF Kanber B Hoskote C Cardoso MJ Ourselin S Duncan JS. Learning to see the invisible: a data-driven approach to finding the underlying patterns of abnormality in visually normal brain magnetic resonance images in patients with temporal lobe epilepsy. Epilepsia. (2019) 60:2499–507. 10.1111/epi.16380 31691273 PMC6972547

